# Combination of epidermal growth factor receptor mutation and the presence of high-grade patterns is associated with recurrence in resected stage I lung adenocarcinoma

**DOI:** 10.1093/icvts/ivac062

**Published:** 2022-03-10

**Authors:** Yasuto Kondo, Junji Ichinose, Hironori Ninomiya, Kohei Hashimoto, Yosuke Matsuura, Masayuki Nakao, Yuichi Ishikawa, Sakae Okumura, Yukitoshi Satoh, Mingyon Mun

**Affiliations:** 1 Department of Thoracic Surgical Oncology, Cancer Institute Hospital, Japanese Foundation for Cancer Research, Tokyo, Japan; 2 Department of Thoracic Surgery, Kitasato University School of Medicine, Kanagawa, Japan; 3 Department of Pathology, Cancer Institute Hospital, Japanese Foundation for Cancer Research, Tokyo, Japan; 4 Division of Pathology, Cancer Institute, Japanese Foundation for Cancer Research, Tokyo, Japan; 5 Department of Pathology, Mita Hospital, International University of Health and Welfare, Tokyo, Japan

**Keywords:** Lung adenocarcinoma, Epidermal growth factor receptor, Recurrence, Surgery, Histological subtype

## Abstract

**OBJECTIVES:**

This study aimed to evaluate the prognostic impact of the combination of epidermal growth factor receptor (EGFR) mutation and the presence of high-grade patterns (solid or micropapillary component) in resected stage I lung adenocarcinoma.

**METHODS:**

Patients who underwent curative resection for pathological stage I lung adenocarcinoma and EGFR mutation analysis were included in this study. The impact of the combination of EGFR mutation and the presence of >5% high-grade patterns on recurrence-free survival (RFS) was retrospectively analysed using Cox proportional hazards model and propensity score-matched analysis.

**RESULTS:**

Among the included 721 patients, EGFR mutations were positive in 380 (52.7%). In the EGFR-mutated group, cases with high-grade patterns showed poorer RFS than those without (5-year RFS, 77.7% vs 92.5%, *P* < 0.001), whereas there were no significant prognostic differences in the EGFR wild-type group (5-year RFS, 89.8% vs 88.2%, *P* = 0.807). Multivariable analyses revealed that the combination of EGFR mutations and the presence of high-grade patterns was associated with poor RFS (hazard ratio = 1.655, *P* = 0.035). Furthermore, EGFR mutation was associated with poor RFS in the group with high-grade patterns (hazard ratio = 2.108, *P* = 0.008). After propensity score matching, EGFR-mutated cases with high-grade patterns showed poorer RFS (*P* = 0.028).

**CONCLUSIONS:**

The combination of EGFR mutation and the presence of high-grade patterns was associated with recurrence in resected stage I lung adenocarcinoma. Histological subtypes, including minor components, should be considered when evaluating the risk of recurrence in patients with EGFR-mutated lung adenocarcinoma.

**Figure:**
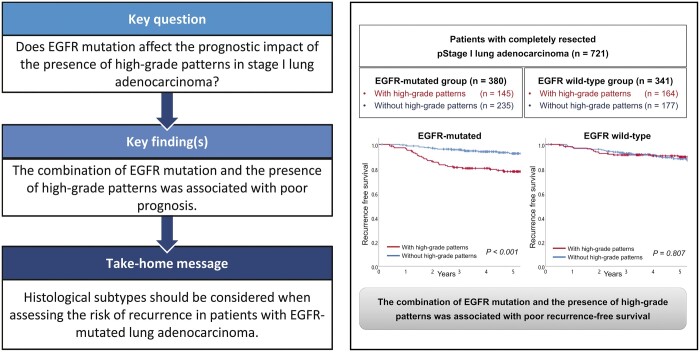

## INTRODUCTION

Lung cancer is the most common cause of cancer-related death worldwide, with 1 378 400 deaths reported annually. Surgical resection with curative intent is considered the standard of care for early-stage non-small-cell lung cancer (NSCLC), and the recurrence rate of stage I NSCLC has been reported to be 12–19% [1–3]. Various factors are known to be risk factors for recurrence, and many papers have reported the importance of histological subtypes in lung adenocarcinoma. The IASLC/ATS/ERS proposed the classification of invasive adenocarcinoma according to the predominant histological subtype after performing comprehensive histological subtyping with semi-quantitative assessment of each subtype in 5% increments [4]. Various studies have reported that solid and micropapillary predominant adenocarcinomas are associated with poor prognosis, placing these subtypes in the high-grade category [5–8]. Moreover, in some studies, adenocarcinomas with >5% solid and micropapillary patterns were associated with poor prognosis even when they were the minor component, and solid and micropapillary components were considered high-grade patterns. Zhao *et al.* [9] reported that the presence of >5% high-grade patterns was associated with higher rates of metastatic lymph nodes and poorer overall survival (OS) and recurrence-free survival (RFS). Yanagawa *et al.* [10] reported that adenocarcinomas with high-grade patterns have poorer OS and RFS. Hung *et al.* [11] also reported that the presence of >5% high-grade patterns was associated with occult mediastinal lymph node metastasis.

Epidermal growth factor receptor (EGFR) mutations are the most common oncogenic driver mutations in lung adenocarcinomas [12, 13]. Several studies have reported the effect of EGFR mutations on prognosis after surgery; however, a consensus has not yet been reached. In a meta-analysis by Zhang *et al.* [14], EGFR mutation positivity was a favourable prognostic factor in terms of OS and RFS. In contrast, another meta-analysis reported by Zhang *et al.* [15] revealed that EGFR mutations were not predictive of OS and RFS.

However, the association between the prognostic influence of high-grade histological subtypes and EGFR mutation status has not been fully investigated. This study aimed to evaluate the prognostic impact of the combination of EGFR mutations and the presence of high-grade patterns in completely resected stage I lung adenocarcinomas.

## MATERIALS AND METHODS

### Ethics statement

This study was approved by the Institutional Review Board for Clinical Research of the Cancer Institute Hospital on 18 March 2021 (approval No. 2020-1321) and was conducted in accordance with the principles of the Declaration of Helsinki and its later amendments. The institutional review board waived the requirement for informed consent due to the retrospective nature of the study.

### Study design and patient cohort

This is a retrospective, observational case–control study. Patients who underwent surgical resection for pathological stage I lung adenocarcinoma at the Cancer Institute Hospital of the Japanese Foundation for Cancer Research from January 2009 to December 2017 were retrospectively included in this study. The exclusion criteria included undergoing sublobar resection, omission of mediastinal lymph node dissection and lack of or inappropriate clinicopathological data.

Using medical records and pathological reports, the following features were reviewed: EGFR mutation status, anaplastic lymphoma kinase (ALK)-rearranged status, age, sex, smoking index (pack-year), tumour size (mm), pleural invasion, lymphovascular invasion, pathological stage, predominant histological subtype, component histological subtype and administration of adjuvant therapy. The pathological staging was based on the 8th edition of the TNM classification for lung cancer [16]. Tumour size was defined as the maximal diameter of the pathological invasive area in the tumour. Pathological evaluation was performed according to the 2015 WHO classification [4]; each histological pattern (lepidic, papillary, acinar, solid, and micropapillary) was recorded in 5% increments. The presence of >5% solid or micropapillary components was defined as high-grade patterns.

Follow-up assessments included physical examination, blood examination including tumour markers and chest and abdominal computed tomography twice annually for 5 years. Patients showing any symptoms or signs of recurrence in these examinations underwent further evaluations, including brain magnetic resonance imaging and positron emission tomography. Recurrence was diagnosed based on the radiological evidence of cancer relapse on surveillance imaging and/or pathological evidence according to tumour biopsy analysis.

### Detection of oncogenic driver mutations

EGFR mutations were evaluated using the Cobas EGFR Mutation Kit v2 (518497453, Roche Diagnostics K.K., Tokyo, Japan), the Scorpion ARMS method (SRL Inc.) or laboratory-developed tests using the peptide nucleic acid-locked nucleic acid polymerase chain reaction clamp method. Genomic DNA was extracted from cubes (3–5 mm^3^) of frozen, fresh lung cancer tissue samples from surgically resected specimens. EGFR point mutations in exon 18 (G719X), exon 20 (S768I) and exon 21 (L858R, L861Q), insertion in exon 20 and deletion in exon 19 were regarded as EGFR mutation positive. The ALK-rearranged status was detected using ALK Detection Kits (Histofine ALK intercalated antibody-enhanced polymer kit, Nichirei Bioscience, Tokyo, Japan). Tumour cells with strongly stained cytoplasm compared to normal cells were defined as positive.

### Statistical analysis

This study aimed to evaluate the prognostic impact of the combination of EGFR mutation and the presence of high-grade patterns. For this purpose, we analysed the association between the combination of EGFR mutation and high-grade patterns with RFS using Cox proportional hazards model. We also evaluated the influence of EGFR mutation on RFS in the subgroup with high-grade patterns using Cox proportional hazards model. The covariates included in the multivariable analyses were age, sex, smoking index, tumour size, pleural invasion and lymphovascular invasion.

Propensity score matching was also applied to adjust for possible confounders between the tw2 groups: EGFR-mutated cases with high-grade patterns and others, which included EGFR mutated without high-grade patterns, and EGFR wild-type cases. Propensity scores were estimated using a logistic regression model including the following associated factors for the combination of EGFR mutation and high-grade patterns: smoking index, tumour size and lymphovascular invasion. Thereafter, the nearest neighbour 1:1 matching method was performed with a calliper of 0.2 standard deviations of the logit of the estimated propensity scores at a ratio of 1:1 without replacement. The balance of the matched cohort was assessed by calculating the standardized difference between the 2 groups; an absolute value of SD >0.1 indicated a meaningful imbalance. RFS estimates of the matched 2 groups were compared by stratified log-rank test with propensity score as a stratification factor.

Continuous variables were expressed using median and interquartile ranges and compared using the Mann–Whitney *U* test, while categorical variables were compared using Fisher’s exact test. OS and RFS were calculated from the date of surgery to the time of death and to the date of relapse or death from any cause, respectively. OS and RFS were estimated using the Kaplan–Meier method and compared using the log-rank test. The reversed Kaplan–Meier method was used to estimate median follow-up duration. Statistical significance was set at *P* < 0.05 in all tests. The criterion for inclusion of the variables in the multivariable models was set at *P* < 0.05 in the univariable analysis. All statistical analyses were performed using EZR (Saitama Medical Center, Jichi Medical University, Saitama, Japan), which is a graphical user interface for R (The R Foundation for Statistical Computing, Vienna, Austria).

## RESULTS

### Patient characteristics

**Figure 1:**
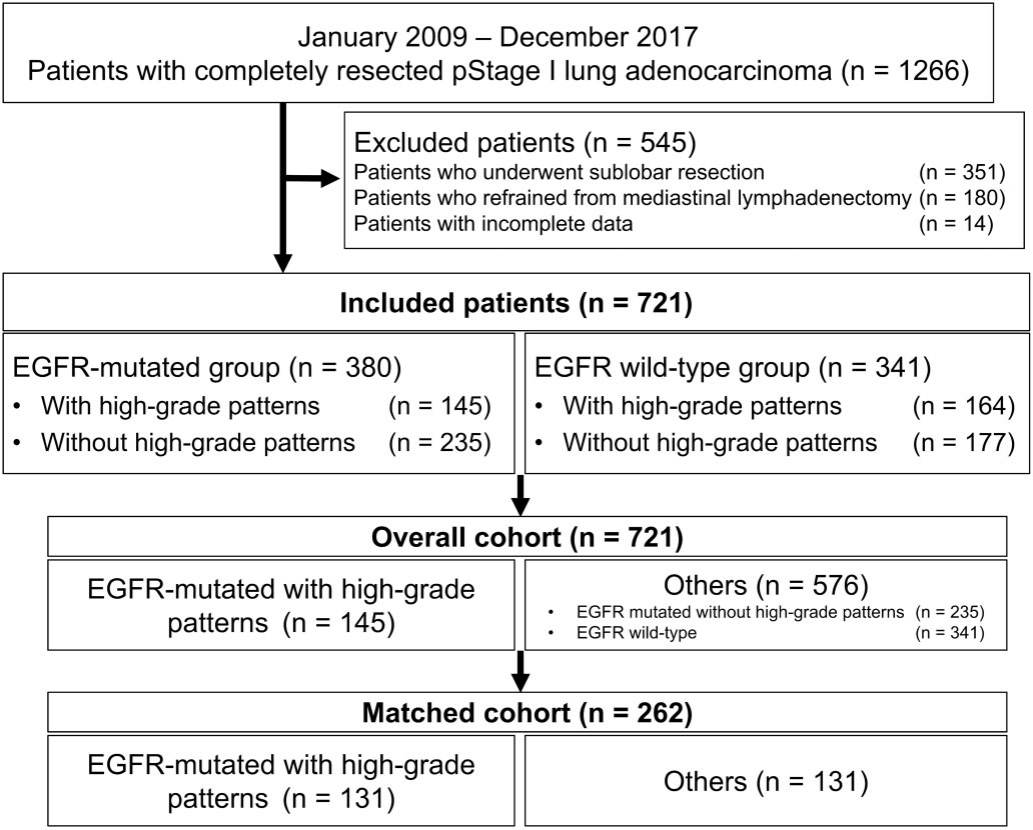
Patient flowchart. EGFR: epidermal growth factor receptor.

After excluding 545 patients (351 underwent sublobar resection, 180 refrained from mediastinal lymphadenectomy and 14 lacked clinicopathological data), a total of 721 patients who underwent curative resection of pathological stage I lung adenocarcinoma were investigated. The study flowchart for patient selection is shown in Fig. 1. Of the 721 patients included in the analysis, 714 underwent lobectomy, 6 underwent bilobectomy and 1 underwent pneumonectomy. Regarding adjuvant therapy, 58 patients received chemotherapy with uracil-tegafur, 2 received chemotherapy with S-1 and 1 received chemotherapy with cisplatin and vinorelbine. No patient underwent adjuvant radiotherapy.

In total, 380 (52.7%) patients harboured EGFR mutations. The EGFR exon 21 L858R point mutation was found in 185 cases, EGFR exon 19 deletion in 168 cases, EGFR exon 18 G719X and exon 21 L861Q mutations in 10 cases each, exon 20 insertion in 5 cases and EGFR 19 mutation and exon 20 S768I point mutations in 1 case each. Ten patients were positive on immunostaining for ALK, and these patients were classified as the EGFR wild-type group.

The association between the EGFR mutation status and clinicopathological features of the 721 cases is shown in Table 1. Positive EGFR mutations were significantly associated with female sex, lower smoking index, absence of pleural and lymphovascular invasion and predominant and component subtypes. In the EGFR-mutated group, 38.1% of patients had high-grade patterns, which was significantly lower than that in the EGFR wild-type group (48.0%). The rate of adjuvant therapy was not significantly different between the EGFR-mutated and wild-type groups.

**Table 1:** Correlation between epidermal growth factor receptor mutation status and clinicopathological features

	EGFR mutated (*n* = 380)	EGFR wild type (*n* = 341)	*P*-Value
Age, years	66 (60, 72)	66 (60, 72)	0.805
Sex, male, *n* (%)	135 (35.5)	186 (54.5)	<0.001
Smoking index, pack-year	0 (0, 8)	17 (0, 40.5)	<0.001
Tumour size, mm	12 (7, 18)	12 (8, 20)	0.256
Pleural invasion, *n* (%)	42 (11.0)	56 (16.4)	0.035
Lymphovascular invasion, *n* (%)	107 (28.2)	134 (39.3)	0.002
Pathological stage, *n* (%)			0.107
IA1	158 (41.5)	125 (36.6)	
IA2	138 (36.3)	114 (33.4)	
IA3	33 (8.6)	36 (10.5)	
IB	51 (13.4)	66 (19.3)	
Predominant subtype, *n* (%)			<0.001
Lepidic	115 (30.2)	83 (24.3)	
Papillary/acinar	252 (66.3)	219 (64.2)	
Solid/micropapillary (high grade)	13 (3.4)	34 (9.9)	
Others	0 (0)	5 (1.4)	
Component subtype,^a^*n* (%)			0.007
Lepidic	368 (96.8)	269 (78.8)	
Papillary/acinar	364 (95.7)	318 (93.2)	
Solid/micropapillary (high grade)	145 (38.1)	164 (48.0)	
Solid	64 (16.8)	80 (23.4)	
Micropapillary	103 (27.1)	117 (34.3)	
Adjuvant therapy, *n* (%)	34 (8.9)	27 (7.9)	0.620

Continuous variables were expressed using median and interquartile range. Stage IA1 was defined as tumour sized 10 mm or less, IA2 was tumour >11 mm but ≤20 mm and IA3 was tumour >21 mm but ≤30 mm. Stage IB was defined as tumour >31 mm but ≤40 mm, or having pleural invasion.

a There can be some extent of subtype overlap in the same patient.

EGFR: epidermal growth factor receptor.

### Prognostic impact of epidermal growth factor receptor mutation and high-grade patterns

**Figure 2:**
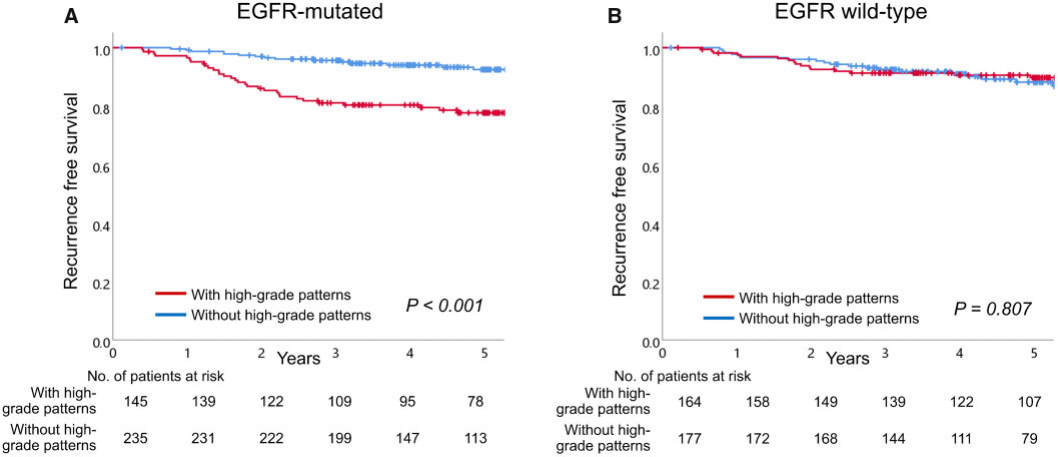
The recurrence-free survival curves according to the presence or absence of high-grade patterns in epidermal growth factor receptor mutated (**A**) and in epidermal growth factor receptor wild type (**B**). EGFR: epidermal growth factor receptor.

The median follow-up time was 5.2 years. Of the 721 patients examined, relapse was observed in 57 (7.9%) cases; 21 patients (2.9%) died due to relapse, whereas 33 patients (4.6%) died due to other causes during follow-up. OS and RFS did not differ between the EGFR mutation-positive and -negative groups (5-year OS, 94.6% vs 93.9%, *P* = 0.592; 5-year RFS, 86.8% vs 89.1%, *P* = 0.759; Supplementary Material, Fig. S1). The association between long-term prognosis and the presence of high-grade patterns was compared according to EGFR mutation status (Fig. 2). In the group with EGFR mutations, cases with high-grade patterns showed poorer RFS than those without (Fig. 2A, 5-year RFS, 77.7% vs 92.5%, respectively, *P* < 0.001), whereas there were no significant prognostic differences in EGFR wild-type cases between groups with and without high-grade patterns (Fig. 2B, 5-year RFS, 89.8% vs 88.2%, respectively, *P* = 0.807).

The associated factors of RFS in stage I lung adenocarcinoma were evaluated by the univariable analysis (Supplementary Material, Table S1). EGFR mutation with high-grade patterns was associated with poor RFS in comparison to EGFR mutations without high-grade patterns and EGFR wild-type cases. Multivariable analysis performed with 6 other factors that were significant in the univariable analysis revealed that the combination of EGFR mutation and the presence of high-grade patterns was associated with poor RFS [hazard ratio (HR) = 1.655, *P* = 0.035], as well as age, tumour size, pleural invasion and lymphovascular invasion (Table 2). We also performed analyses in the subgroup with high-grade patterns. The patient characteristics were compared using EGFR mutation among cases with high-grade patterns (Supplementary Material, Table S2). Although the influence of EGFR mutation on RFS was not significant in the total population (Supplementary Material, Table S1), in the group with high-grade patterns, univariable and multivariable analyses showed that EGFR mutation was significantly associated with poor RFS (HR = 2.108, *P* = 0.008; Table 3).

**Table 2:** The associated factors of recurrence-free survival in stage I lung adenocarcinoma evaluated by multivariable analysis

	HR (95% CI)	*P*-Value
EGFR mutated with high-grade patterns	1.655 (1.037–2.643)	0.035
Age (years)	1.029 (1.004–1.055)	0.023
Male	1.446 (0.895–2.336)	0.132
Smoking index (pack-year)	0.998 (0.989–1.007)	0.699
Tumour size (mm)	1.040 (1.015–1.066)	0.002
Pleural invasion	2.600 (1.610–4.199)	< 0.001
Lymphovascular invasion	1.892 (1.165–3.075)	0.010

The effect of variables on RFS was analysed by multivariable analysis using a Cox proportional hazards model. The HR of EGFR mutated with high-grade patterns was calculated in comparison with EGFR mutated without high-grade patterns and EGFR wild-type cases.

CI: confidence interval; EGFR: epidermal growth factor receptor; HR: hazard ratio; RFS: recurrence-free survival.

**Table 3:** The effect of epidermal growth factor receptor mutation on recurrence-free survival in the group with high-grade patterns

	Univariable		Multivariable	
	HR (95% CI)	*P*-Value	HR (95% CI)	*P*-Value
EGFR mutation	1.752 (1.014–3.028)	0.044	2.108 (1.213–3.665)	0.008
Age (years)	1.035 (1.002–1.070)	0.037	1.041 (1.007–1.075)	0.017
Male	1.252 (0.718–2.183)	0.428		
Smoking index (pack-year)	1.000 (0.991–1.010)	0.922		
Tumour size (mm)	1.059 (1.028–1.091)	< 0.001	1.040 (1.006–1.074)	0.019
Pleural invasion	3.884 (2.259–6.679)	< 0.001	2.928 (1.644–5.215)	<0.001
Lymphovascular invasion	3.575 (1.745–7.325)	< 0.001	2.899 (1.384–6.075)	0.005

The effect of variables on RFS was analysed by univariable and multivariable analyses using a Cox proportional hazards model.

CI: confidence interval; EGFR: epidermal growth factor receptor; HR: hazard ratio; RFS: recurrence-free survival.

### Propensity score-matched analysis

**Figure 3:**
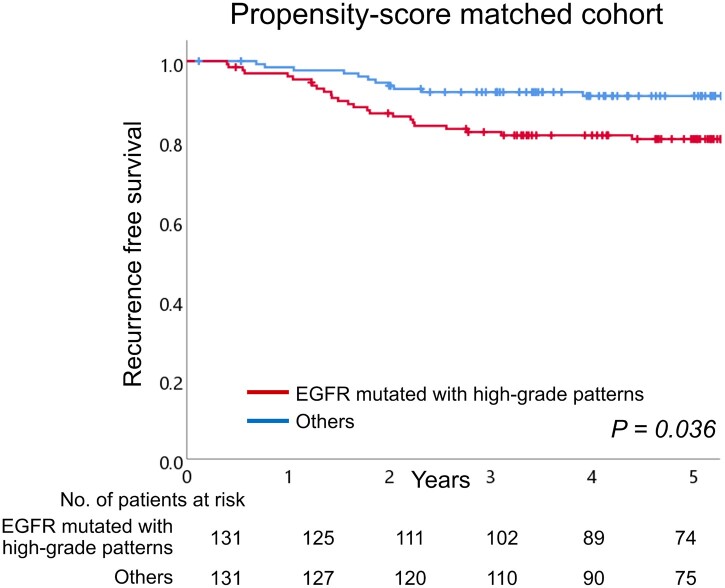
The recurrence-free survival curves of epidermal growth factor receptor mutated with high-grade patterns and others (epidermal growth factor receptor mutated without high-grade patterns and epidermal growth factor receptor wild-type cases) in the propensity score-matched cohort. EGFR: epidermal growth factor receptor.

Furthermore, propensity score-matched analysis was performed between EGFR mutations with high-grade patterns and others (EGFR mutations without high-grade patterns and EGFR wild-type cases). Propensity scores were estimated using a logistic regression model including the following associated factors for the combination of EGFR mutation and high-grade patterns: smoking index, tumour size and lymphovascular invasion (Supplementary Material, Table S3). After propensity score matching, the imbalance of these confounding factors was appropriately adjusted for (Table 4). EGFR-mutated cases with high-grade patterns showed poorer RFS than others in the matched cohort (5-year RFS, 80.4% vs 91.2%; Fig. 3). A stratified log-rank test showed that the difference in survival rates was significant (*P* = 0.028).

**Table 4:** Associated factors for the combination of epidermal growth factor receptor mutation and high-grade patterns before and after matching

	Overall cohort (*n* = 721)			Matched cohort (*n* = 262)		
	EGFR mutated with high-grade patterns (*n* = 145)	Others (*n* = 576)	SD	EGFR mutated with high-grade patterns (*n* = 131)	Others (*n* = 131)	SD
Smoking index, pack-year	0 (0, 15)	0 (0, 30)	0.351	0 (0, 15)	0 (0, 15)	0.025
Tumour size, mm	16 (11, 20.5)	11 (7, 18)	0.472	15 (11, 20)	15 (11, 21)	0.020
Lymphovascular invasion, *n* (%)	79 (54.5)	162 (28.1)	0.556	66 (50.4)	66 (50.4)	<0.001

Continuous variables were expressed using median and interquartile range. Others included EGFR-mutated without high-grade patterns and EGFR wild-type cases.

EGFR: epidermal growth factor receptor; SD: standardized difference.

## DISCUSSION

In our study, EGFR mutation was significantly associated with poor RFS of stage I lung adenocarcinoma only in cases with high-grade patterns. Multivariable analysis using Cox proportional hazard model and propensity score-matched analysis revealed that the combination of EGFR mutation and the presence of high-grade patterns was associated with poor RFS in resected stage I lung adenocarcinoma.

Among the histological subtypes of adenocarcinoma, many studies have confirmed that the solid and micropapillary predominant subtypes are associated with high malignancy and poor prognosis [5–8], and this effect has been reported to be seen even when they are the minor component [9–11]. The presence of micropapillary pattern increases the ability of cancer cells to invade blood vessels, and 55% of patients with lung adenocarcinoma with micropapillary pattern have been reported to have vascular invasion [17]. It has also been shown that a >5% micropapillary pattern is associated with a higher risk of recurrence in patients who underwent sublobar resection compared to those who underwent lobectomy [18]. In early-stage lung adenocarcinoma, the number of circulating endothelial progenitor cells differed according to histological subtype and was reported to be the highest in the solid type [19]. Two reports investigating patients who underwent resection for EGFR-mutated lung cancer showed that the incidence of brain metastasis was higher and the time for development of brain metastasis was shorter in patients with micropapillary patterns [20], whereas patients with solid patterns had a higher incidence of bone metastasis and a shorter time of development of bone metastasis [21].

However, few studies have examined the association between the prognostic impact of high-grade histologic subtypes and the presence of EGFR mutations. Recently, results of a multicentre study revealed that EGFR mutation was a risk factor for recurrence when high-grade histological subtype was predominant [22]. In stage I high-grade predominant cases and stage II-IIIA cases, EGFR mutation-positive cases showed poorer RFS than EGFR mutation-negative cases (5-year RFS, 49.6% vs 75.6%, respectively, *P* < 0.01), whereas there was no significant difference in RFS according to the EGFR mutation status in stage I low- and intermediate-grade predominant cases. Positive EGFR mutations were significantly associated with poorer RFS in stage I high-grade predominant cases and stage II-IIIA cases (HR = 2.005, *P* = 0.041), which was similar to the results of the present study. These data are highly interesting, but the low frequency of high-grade predominant cases poses a problem. Ito *et al.* [22] reported that only 13 (1.1%) of the 1155 investigated cases had EGFR mutations and a high-grade predominant subtype. The same is true for the patients included in this study, where the combination of an EGFR mutation and a high-grade predominant subtype was detected in only 13 cases (1.8%). Therefore, we focused on adenocarcinomas with >5% solid or micropapillary components, even though they were not predominant. The combination of an EGFR mutation and the presence of a high-grade pattern was detected in 145 cases, accounting for 20.1% of the total cohort. This classification is more useful for assessing the risk of recurrence in daily clinical practice.

It is important to identify the subgroup with a high risk of recurrence in patients with stage I NSCLC when considering adjuvant chemotherapy because the postoperative recurrence rate of early-stage lung cancer is low. Adjuvant cisplatin-based chemotherapy is recommended for patients with completely resected stage II to IIIA NSCLC. This therapy is associated with a 16% decrease in the risk of disease recurrence or death [23]. Kato *et al.* [24] reported that adjuvant chemotherapy with uracil-tegafur improved the survival rates of patients with stage I NSCLC measuring 2 cm or more in diameter. Recently, the usefulness of EGFR-tyrosine kinase inhibitors in adjuvant chemotherapy has been reported. Zhong *et al.* [25] reported that the median RFS was significantly longer in the gefitinib group than in the vinorelbine plus cisplatin group among patients with resected stage II to IIIA NSCLC with EGFR mutations (median RFS, 30.8 vs 19.8 months, respectively, *P* = 0.001). Wu *et al.* [26] reported that RFS was significantly longer among those who received osimertinib than among those who received placebo in patients with stage IB–IIIA EGFR mutation-positive NSCLC. When considering adjuvant therapy using EGFR-tyrosine kinase inhibitors for stage I cases, the evaluation of the risks and benefits is necessary. Our study demonstrated that EGFR mutation-positive cases with high-grade patterns have a relatively higher risk of recurrence among stage IA–IB adenocarcinomas. Even in stage IA adenocarcinomas, it is possible that this group may be candidates for adjuvant chemotherapy using EGFR-tyrosine kinase inhibitors.

### Limitations

This study has some limitations. First, it was a retrospective single-centre study and most of the patients were East Asians; hence, our conclusions may not be extrapolated to the global population. Second, there may have been some selection bias. This study was limited to patients who underwent lobectomy with mediastinal lymph node dissection. Patients who underwent sublobar resection or limited lymph node dissection due to radiological features or underlying conditions were excluded. Third, EGFR mutations were detected by different methods, which may have led to heterogeneous accuracy.

## CONCLUSION

EGFR mutation was significantly associated with poor RFS of stage I lung adenocarcinoma only in cases with high-grade patterns. The combination of EGFR mutation and the presence of high-grade patterns was associated with recurrence in resected stage I lung adenocarcinoma. Histological subtypes, including minor components, should be considered when evaluating the risk of recurrence in patients with EGFR-mutated lung adenocarcinoma.

## SUPPLEMENTARY MATERIAL

Supplementary material is available at *ICVTS* online.

## Supplementary Material

ivac062_Supplementary_Data

## Notes

Presented at the IASLC 2021 World Conference on Lung Cancer, September 2021, virtual meeting.

## ABBREVIATIONS

ALK: Anaplastic lymphoma kinase
EGFR: Epidermal growth factor receptor
HR: Hazard ratio
NSCLC: Non-small cell lung cancer
OS: Overall survival
RFS: Recurrence-free survival
