# Supplementary material for: Combination of epidermal growth factor receptor mutation and the presence of high-grade patterns is associated with recurrence in resected stage I lung adenocarcinoma
Source: Interact Cardiovasc Thorac Surg. 2022 Mar 10;35(2):ivac062. doi: 10.1093/icvts/ivac062 (PMC9297517; doi:10.1093/icvts/ivac062)
Supplement: ivac062_Supplementary_Data [file ivac062_supplementary_data.zip › supplementary materials.docx]

**Supplementary Table S1.** The associated factors of RFS in stage I lung adenocarcinoma evaluated by univariable analysis.

|  | HR (95% CI) | P-value |
| --- | --- | --- |
| EGFR-mutated with high-grade patterns | 2.215 (1.434–3.421) | < 0.001 |
| EGFR mutation | 1.067 (0.705–1.614) | 0.759 |
| High-grade patterns | 1.885 (1.238–2.870) | 0.003 |
| Age (year) | 1.027 (1.001–1.054) | 0.038 |
| Male | 1.780 (1.169–2.708) | 0.007 |
| Smoking index (pack-year) | 1.007 (1.000–1.014) | 0.042 |
| Tumour size (mm) | 1.070 (1.047–1.092) | < 0.001 |
| Pleural invasion | 4.500 (2.926–6.922) | < 0.001 |
| Lymphovascular invasion | 3.492 (2.280–5.348) | < 0.001 |

RFS, recurrence-free survival; HR, hazard ratio; CI, confidence interval; EGFR, epidermal growth factor receptor.

The associated factors with RFS were evaluated by univariable analysis using a Cox proportional hazards model.

The HR of EGFR-mutated with high-grade patterns was calculated in comparison with EGFR-mutated without high-grade patterns and EGFR wild-type cases.

**Supplementary Table S2.**

Association between EGFR mutation status and clinicopathological features in the subgroup with high-grade patterns.

|  | EGFR-mutated with high-grade patterns  (n = 145) | EGFR wild-type with high-grade patterns  (n = 164) | *P*-value |
| --- | --- | --- | --- |
| Age, year | 66 (59, 72) | 65.5 (60, 71.75) | 0.611 |
| Sex, male (%) | 65 (44.8%) | 109 (66.5%) | < 0.001 |
| Smoking index, pack-year | 0 (0, 15) | 27 (3, 47) | < 0.001 |
| Tumour size, mm | 16 (11, 20.5) | 15 (11, 23) | 0.982 |
| Pleural invasion (%) | 33 (22.8%) | 43 (26.2%) | 0.481 |
| Lymphovascular invasion (%) | 79 (54.5%) | 105 (64.0%) | 0.088 |
| Pathological stage |  |  | 0.454 |
| IA1 (%) | 26 (17.9%) | 32 (19.5%) |  |
| IA2 (%) | 64 (44.1%) | 58 (35.4%) |  |
| IA3 (%) | 19 (13.1%) | 24 (14.6%) |  |
| IB (%) | 36 (24.8%) | 50 (30.5%) |  |
| Adjuvant therapy (%) | 24 (16.6%) | 23 (14.0%) | 0.537 |

EGFR, epidermal growth factor receptor.

Continuous variables were expressed using median and interquartile range.

**Supplementary Table S3.** Associated factors for EGFR mutations with high-grade patterns

|  | Odds ratio (95% CI) | P-value |
| --- | --- | --- |
| Age (year) | 1.005 (0.984–1.025) | 0.670 |
| Male | 1.456 (0.929–2.282) | 0.101 |
| Smoking index (pack-year) | 0.968 (0.955–0.980) | < 0.001 |
| Tumour size (mm) | 1.037 (1.013–1.063) | 0.003 |
| Pleural invasion | 1.318 (0.766–2.266) | 0.318 |
| Lymphovascular invasion | 3.078 (2.000–4.736) | < 0.001 |

EGFR, epidermal growth factor receptor; CI, confidence interval.

Associated factors for EGFR-mutated with high-grade patterns were analysed by multivariable analysis using logistic regression analysis.

**Supplementary Figure S1**

The overall survival curves (A) and recurrence-free survival curves (B) according to EGFR mutation status in resected stage I lung adenocarcinoma.

EGFR, epidermal growth factor receptor
