# Supplementary figures and images for: Combination of epidermal growth factor receptor mutation and the presence of high-grade patterns is associated with recurrence in resected stage I lung adenocarcinoma
Source: Interact Cardiovasc Thorac Surg. 2022 Mar 10;35(2):ivac062. doi: 10.1093/icvts/ivac062 (PMC9297517; doi:10.1093/icvts/ivac062)

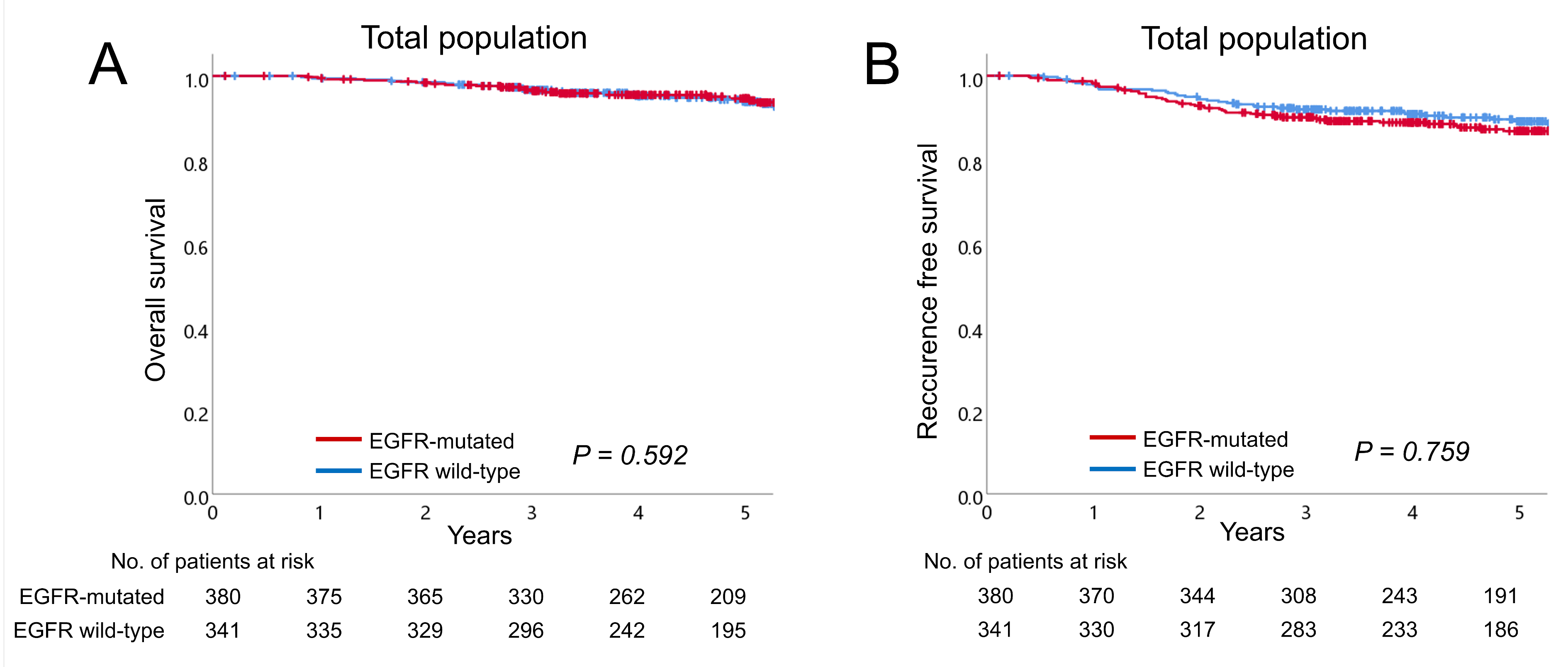

Supplement: ivac062_Supplementary_Data [file ivac062_supplementary_data.zip › Figure S1.tif]
